# The association between use of statin or aspirin and pancreatic ductal adenocarcinoma: A nested case‐control study in a Korean nationwide cohort

**DOI:** 10.1002/cam4.2617

**Published:** 2019-10-21

**Authors:** Jin Ho Choi, Sang Hyub Lee, Gunn Huh, Jung Won Chun, Min Su You, Woo Hyun Paik, Ji Kon Ryu, Yong‐Tae Kim

**Affiliations:** ^1^ Department of Internal Medicine Liver Research Institute Seoul National University College of Medicine Seoul Korea

**Keywords:** aspirin, cohort, pancreatic cancer, prevention, statin

## Abstract

**Background:**

Although several studies have suggested that aspirin and statins may help prevent pancreatic ductal adenocarcinoma (PDAC), this concept has been controversial. This study aimed to evaluate the association between use of statin or aspirin and PDAC in a nationwide large cohort.

**Methods:**

In this nested case‐control study, we used data from a 12‐year nationwide longitudinal cohort in Korea. Cases with PDAC and controls who were matched to cases by age, sex, income, and index year at a 1:5 ratio were established. We used multivariate logistic regression analyses to identify the independent risk factors of PDAC.

**Results:**

We identified a total of 827 patients with PDAC between 2007 and 2013, and included 4135 matched controls. Diabetes mellitus, chronic and acute pancreatitis, pancreatic cystic lesions, and cholelithiasis were independent risk factors for PDAC. Statin use (odds ratio [OR], 0.92; 95% confidence interval [CI] 0.76‐1.11; *P* = .344; adjusted OR [aOR], 0.70; 95% CI 0.56‐0.87; *P* = .001) was associated with a reduced risk of PDAC after correction of the confounding factors, but aspirin use (OR, 0.98; 95% CI 0.84‐1.15; *P* = .838; aOR 0.84; 95% CI 0.70‐1.01, *P* = .068) was not associated with PDAC. Among the patients with risk factors, both statin use (OR, 0.50; 95% CI 0.38‐0.66; *P* < .001; aOR, 0.62; 95% CI 0.45‐0.84; *P* = .002) and aspirin use (OR, 0.48; 95% CI 0.31‐0.67; *P* < .001; aOR 0.67; 95% CI 0.50‐0.89, *P* = .006) were associated with a reduced risk of PDAC.

**Conclusion:**

This study suggests that statin use was associated with a reduced risk of PDAC incidence but aspirin use was not. Both statin use and aspirin use were associated a reduced risk of PDAC incidence for patients with risk factors.

## Graphical abstract

The main contribution of our paper is to identify the association between use of statin or aspirin and pancreatic cancer in a nationwide large cohort, which is one of the worst prognostic cancers. In this nested case‐control study in the Korean nationwide cohort, we found that statin use was associated with a reduced risk of pancreatic cancer incidence but aspirin use was not. Both statin use and aspirin use were associated a reduced risk of pancreatic cancer incidence for patients with risk factors of pancreatic cancer.

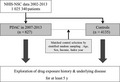

## 1 INTRODUCTION

Pancreatic ductal adenocarcinoma (PDAC) is one of the worst prognostic cancers, with an estimated overall 5‐year survival rate under 10% despite advances in treatments leading to better clinical outcomes, and it is expected to be the second‐most leading cause of death by 2020.^1^, ^2^ Approximately 90% of pancreatic cancers are sporadic, and several non‐genetic risk factors have been reported as follows: Tobacco exposure, diabetes mellitus (DM), chronic pancreatitis (CP), acute pancreatitis (AP), gallstones, pancreatic mucinous cystic lesions, and pancreatic intraepithelial neoplasm.^3^, ^4^, ^5^, ^6^ It is important to look for the risk factors of PDAC because it tends to be diagnosed as an advanced disease, but there is no impressive progression even though many studies including chemoprevention have been done.^3^, ^7^

Recent studies have suggested the additive cancer‐prevention effects of drugs, including statin and aspirin, whose mechanism of action is well understood and whose safety has been guaranteed by widespread usage for a long time. First, several researchers have suggested that statin has a cancer‐prevention effect, and the mechanism is suggested as follows: suppression of the mevalonate pathway,^8^ inhibition of tumor growth and metastasis by reducing inflammation,^9^ antiangiogenic effects,^10^ and inhibition of proliferation and triggering apoptosis of tumor cells.^11^ Nonetheless, the results of the clinical studies are reported to vary widely. One meta‐analysis^12^ insists that statins still have a protective effect on pancreatic cancer, but two other meta‐analyses denied the effect.^13^, ^14^ Therefore, the anticancer effect of statins is also not clear yet.

Various studies have reported on the anticancer effect of aspirin. Researchers have suggested that aspirin can help prevent and treat cancer.^15^ Several studies have proposed an explanation for the antitumor effect of aspirin, which is given as follows: cell apoptosis by inhibiting Bcl‐2 expression and down regulating cyclooxygenase‐2 expression,^16^, ^17^ improving DNA self‐healing,^18^ and controlling the number of circulating platelets and their activity levels to evade several innate antitumor effects.^19^ The anticancer effect of aspirin for pancreatic cancer is not clear, because clinical studies on pancreatic cancer have conflicting results.^20^, ^21^

We conducted this nested case‐control study in the National Health Insurance Services‐National Sample Cohort (NHIS‐NSC), to identify the association between use of statin or aspirin and PDAC.

## 2 MATERIAL AND METHODS

### 2.1 Study population and outcomes

This study was exempt from ethics review by the institutional review board because the data originated from deidentified secondary data released by the National Health Insurance Service (NHIS) for research purposes. All health‐care providers need to submit medical claims to NHIS for review and reimbursement, with data including information on demographics, diagnostic codes, and prescription records. The NHIS‐NSC included claims submitted by health‐care providers from 2002 through 2013, which were provided to researchers for research purposes. The total population of this cohort consisted of 1 025 340 nationally representative random subjects, which represents approximately 2.2% of the total number of patients enrolled in NHIS in 2002. We selected patients using the stratified random sampling method, with 1476 strata according to sex, age, and income level.^22^

The primary outcome of this study was to evaluate the association between statin or aspirin use and PDAC. The secondary outcomes were to identify any dose‐effect association between each drug and PDAC, and to evaluate risk factors of PDAC.

### 2.2 The operational definition of PDAC and other conditions

We collected PDAC patients since 1 January 2007 to analyze their medication exposure and comorbidities for at least 5 years for each patient. We identified newly diagnosed PDAC cases between 1 January 2007, and 31 December 2013, by following criteria: (a) hospitalization with the International Classification of Diseases Tenth Revision (ICD‐10) code C25 (except C254) at least once; (b) Underwent one or more imaging tests including computed tomography, magnetic resonance imaging, abdominal ultrasonography, and endoscopic ultrasound; and (c) Visit an outpatient clinic related to the code three or more times. The accuracy of this operational definition of PDAC was demonstrated in a previous Korean study which compared the cancer incidence rates between the National Cancer Registry and insurance claims data.^23^ The index date was defined as the first date of the PDAC diagnosis. The patients who were diagnosed with any other cancer prior to the index date for PDAC group were excluded. Also, to ensure that all study subjects in the PDAC group and the control group had at least 1 year of drug‐free period, subjects who were prescribed statins or aspirin in 2002 were excluded. We included control subjects without PDAC, matched by age, sex, income level, and the index year to cases at a 1:5 ratio.

We additionally confirmed the following operational definitions for several comorbidities according to the corresponding ICD‐10 code. The presence of DM was identified by having a prescription for antidiabetic drugs with the DM code (E10‐14). Chronic pancreatitis was defined as the code of K86.0 and K86.1 with any preceding image tests. Acute pancreatitis was defined as the code of K85 at hospitalization with any preceding image tests. Cholelithiasis was defined as the code of K80 with any preceding image tests. Pancreatic cystic lesion (PCL) was defined as the code of K86.2 and D13.6 with any preceding image tests. We also identified alcohol‐related disease as alcoholic liver disease (K70) with any preceding image tests and mental and behavioral disorders due to use of alcohol (F10). In addition, we examined chronic obstructive pulmonary disease (COPD; J43, J44) with medications for COPD as representative smoking‐related diseases.^24^ Chronic hepatitis B (B18.0, B18.1) chronic hepatitis C (B18.2), and non‐alcoholic fatty‐liver disease (K76.0) were also evaluated.

### 2.3 Information on drug exposure

Drug exposure was defined as a prescription over 30 defined daily dose (DDD) in outpatient visits from 2002 to the index date, and we applied a lag period of 6 months to allow a reasonable induction period for a drug effect to occur and to preclude reverse causation.^25^ We also calculated cumulative exposure for drugs with the sum of the doses for all of the prescribed days which was described as the cumulative DDD. We used the DDD system as defined by the World Health Organization Collaborating Center for Drug Statistics Methodology.^26^ Drug exposure of aspirin was measured by DDD of low‐dose aspirin and that of statins was calculated by DDD of each drug used in dyslipidemia. Cumulative exposure was described in the unit of DDD‐year, which means 365 DDDs. In this study, the aspirin included aspirin enteric coated, and aspirin encapsulated, and the statins included atorvastatin, fluvastatin, lovastatin, pitavastatin, pravastatin, rosuvastatin, and simvastatin.

### 2.4 Statistical analyses

The baseline characteristics were compared between the PDAC and control group using *t* tests for continuous variables and the chi‐squared test with Fisher's exact test for categorical variables. We used univariate and multivariate logistic regression to compute the odds ratio (OR) and adjusted OR (aOR) with 95% confidence intervals (CI) for the association between pancreatic cancer and drug exposure. For the sensitivity analyses, we conducted additional analysis for exposure to each drug by applying three different lag times (6 months, 1 month, and 1 year). We conducted the analyses to evaluate the dose‐effect relationship compared with the unexposed patients by using the following criteria for amount of drug exposure calculated using DDD by applying the lag time of 6 months: (a) two groups divided by the median value of drug exposure; (b) three groups divided by 0.5 DDD‐year interval for statin and one DDD‐year interval for aspirin.

We further analyzed to find the other risk factors for PDAC other than age, sex, income, and index year, because we had already matched them at the control selection step. Suspected confounding factors for development of PDAC were adjusted by multivariate logistic regression in the analyses of drug exposure. In addition, we performed a subgroup analysis to identify the correlation of statin use or aspirin use and risk of PDAC incidence in patients with risk factors.

SAS version 9.4 (SAS Institute, Inc) was used for statistical analysis. *P* value <.05 was considered to be statistically significant.

## 3 RESULTS

### 3.1 Baseline characteristics of the patients in this study

**Figure 1:**
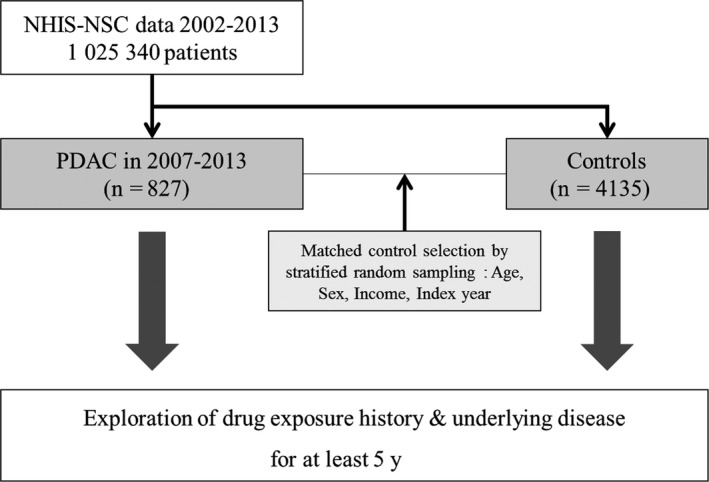
Flowchart of this study

In this study, 827 patients with newly developed PDAC were identified during the period 2007‐2013, and we examined 4135 patients without PDAC and sociodemographically matched control subjects (Figure 1). The baseline characteristics of the PDAC group and the control group are shown in Table 1. Age, sex, income, and year of the index date were similar in both groups. There were 41.8% male in each group, and about half the patients in each group were over 70 years old. The proportion of individuals who had DM, AP, CP, PCL, and cholelithiasis was higher in the PDAC group than in the control group. Meanwhile, the proportion of individuals who had alcohol‐related disease, COPD, chronic hepatitis B infection, and chronic hepatitis C infection were not different between the two groups.

**Table 1:** Baseline characteristics of the patients in this study

	PDAC group (N = 827)		Control group (N = 4135)		*P*
	n	%	n	%	
Age group, y					
<40	8	1.0	40	1.0	1.000
40‐49	48	5.8	240	5.8	
50‐59	129	15.6	645	15.6	
60‐69	223	27.0	1115	27.0	
Over 70	419	50.7	2095	50.7	
Sex					
Male	346	41.8	1730	41.8	1.000
Female	481	58.2	2405	58.2	
Income level					
Low	258	31.2	1290	31.2	1.000
Intermediate	186	22.5	930	22.5	
High	383	46.3	1915	46.3	
Index date					
2007‐2009	324	39.2	1620	39.2	1.000
2010‐2013	503	61.8	2515	61.8	
Comorbidity					
DM	257	31.1	700	16.9	<.001
AP	87	10.5	30	0.7	<.001
CP	75	9.1	7	0.2	<.001
PCL	74	8.9	7	0.2	<.001
Cholelithiasis	71	8.6	123	3.0	<.001
Alcohol‐related disease	77	9.3	358	8.7	.624
ALD	66	7.9	287	6.9	.299
Alcohol use disorder	11	1.3	71	1.7	.549
COPD	29	3.5	171	4.1	.439
CHB	33	3.9	132	3.2	.243
CHC	14	1.7	46	1.1	.164

Abbreviations: ALD, alcoholic liver disease; AP, acute pancreatitis; CHB, chronic hepatitis B infection; CHC, chronic hepatitis C infection; COPD, chronic obstructive pulmonary disease; CP, chronic pancreatitis; DM, diabetes mellitus; IPF, idiopathic pulmonary fibrosis; PCL, pancreatic cystic lesion; PDAC, pancreatic ductal adenocarcinoma.

### 3.2 Risk factors of PDAC

Table 2 shows the univariate and multivariate logistic regression analysis of risk factors for PDAC. Diabetes mellitus, AP, CP, PCL, and cholelithiasis showed a significant difference between the PDAC group and the control group. In multivariate logistic regression analysis, the aspirin model was adjusted for aspirin ever user and the statin model was adjusted for statin ever user. Chronic pancreatitis (aOR in aspirin model, 48.79 [95% CI 21.96‐108.38; *P* < .001]; aOR in statin model, 49.69 [95% CI 22.33‐110.55; *P* < .001]) and PCL (aOR in aspirin model, 43.08 [95% CI 19.31‐96.12; *P* < .001]; aOR in statin model, 44.14 [95% CI 19.76‐98.60; *P* < .001]) were revealed as the most obvious risk factors for PDAC. AP (aOR in aspirin model, 13.37 [95% CI 8.54‐20.94; *P* < .001]; aOR in statin model 13.52 [95% CI 8.63‐21.17; *P* < .001]) was also found to be a strong risk factor, followed by cholelithiasis (aOR in aspirin model, 2.72 [95% CI 1.94‐3.81, *P* < .001]; aOR in statin model, 2.69 [95% CI 1.92‐3.78; *P* < .001]) and DM (aOR in aspirin model, 2.29 [95% CI 1.89‐2.78; *P* < .001]; aOR in statin model 2.38 [95% CI 1.96‐2.89; *P* < .001]). Alcohol‐related disease, COPD, and viral chronic hepatitis were not evaluated as risk factors for PDAC.

**Table 2:** Univariate and multivariate logistic regression analysis of risk factors for PDAC

Factors	Univariate		Multivariate		Multivariate	
			Aspirin model^a^		Statin model^b^	
	OR (95% CI)	*P*‐value	aOR (95% CI)	*P*‐value	aOR (95% CI)	*P*‐value
DM	2.21 (1.87‐2.62)	<.001	2.29 (1.89‐2.78)	<.001	2.38 (1.96‐2.89)	<.001
AP	16.09 (10.55‐24.54)	<.001	13.37 (8.54‐20.94)	<.001	13.52 (8.63‐21.17)	<.001
CP	58.52 (27.00‐128.11)	<.001	48.79 (21.96‐108.38)	<.001	49.69 (22.33‐110.55)	<.001
PCL	57.95 (26.60‐126.29)	<.001	43.08 (19.31‐96.12)	<.001	44.14 (19.76‐98.60)	<.001
Cholelithiasis	3.06 (2.26‐4.15)	<.001	2.72 (1.94‐3.81)	<.001	2.69 (1.92‐3.78)	<.001
Alcohol‐related disease	1.07 (0.82‐1.40)	.62				
COPD	0.87 (0.59‐1.28)	.51				
CHB	1.26 (0.85‐1.86)	.24				
CHC	1.53 (0.84‐2.80)	.16				

Abbreviations: aOR, adjusted odds ratio; AP, acute pancreatitis; CHB, chronic hepatitis B infection; CHC, chronic hepatitis C infection; CI confidence interval; CP, chronic pancreatitis; DM, diabetes mellitus; OR, odds ratio; PCL, pancreatic cystic lesion; PDAC, pancreatic ductal adenocarcinoma.

a Adjusted age, sex, income, index year and underlying disease including DM, PCL, AP, CP, cholelithiasis and aspirin use.

b Adjusted age, sex, income, index year and underlying disease including DM, PCL, AP, CP, cholelithiasis and statin use.

### 3.3 Association between statin use and PDAC

In this study, 19.1% (158 of 827) of the patients in the PDAC group and 20.6% (851 of 4135) of the patients in the control group had ever used statin. (*P* = .344). The median statin exposure was 0.91 DDD‐years (IQR, 0.32‐2.20) in 1009 patients (Table 3). We also calculated the aORs with 95% CIs of the association between exposure to statin and PDAC with adjustment of confounding factors, including DM, AP, CP, PCL, and cholelithiasis. The analyses of statin exposure showed a significant difference between the two groups after correcting the confounding factors. The OR was 0.92 (95% CI 0.76‐1.11; *P* = .344) and the aOR was 0.70 (95% CI 0.56‐0.87; *P* = .001). These results were observed to be the same when different lag times were applied. The dose‐effect relationship analysis of statin by DDD‐year showed that the OR was 0.86 (95% CI 0.66‐1.11; *P* = .25) and the aOR was 0.67 (95% CI 0.50‐0.90; *P* = .008) for a “less than median” user, as the OR was 0.97 (95% CI 0.75‐1.24, *P* = .78) and the aOR was 0.72 (95% CI 0.54‐0.95; *P* = .022) for the “more than median” user. The ORs and aORs of three groups divided by 0.5 DDD‐year interval were as follows: 0.85 (95% CI 0.63‐1.15; *P* = .29) and 0.69 (95% CI 0.49‐0.98; *P* = .038) for a user of less than 0.5 DDD‐year, 0.86 (95% CI 0.57‐1.31; *P* = .48) and 0.60 (95% CI 0.37‐0.97; *P* = .037) for a user for 0.5 to one DDD‐year 0.98 (95% CI 0.76‐1.26; *P* = .87) and 0.73 (95% CI 0.55‐0.98, *P* = .036) for a user for more than one DDD‐year.

**Table 3:** Relationship of statin use to PDAC

	LT	Dose (DDD‐y)	PDAC	Control	Crude		Adjusted^a^	
			(n = 827) N (%)	(n = 4135) N (%)	OR (95% CI)	*P*	OR (95% CI)	*P*
Statin use								
Never	6 mo		669 (80.9)	3284 (79.4)				
Ever	6 mo		158 (19.1)	851 (20.6)	0.92 (0.76‐1.11)	.344	0.70 (0.56‐0.87)	.001
Never	1 mo		669 (80.9)	3284 (79.4)				
Ever	1 mo		158 (19.1)	851 (20.6)	0.92 (0.76‐1.11)	.344	0.70 (0.56‐0.87)	.001
Never	1 y		679 (82.1)	3331 (80.6)				
Ever	1 y		148 (17.9)	804 (19.4)	0.90 (0.74‐1.10)	.310	0.70 (0.56‐0.88)	.002
Cumulative dose of statin use								
Never	6 mo		669 (80.9)	3284 (79.4)	1.00		1.00	
Ever	6 mo							
		Less than median (<0.91)	75 (9.1)	429 (10.4)	0.86 (0.66‐1.11)	.25	0.67 (0.50‐0.90)	.008
		More than median (≥0.91)	83 (10.0)	422 (10.2)	0.97 (0.75‐1.24)	.78	0.72 (0.54‐0.95)	.022
Cumulative dose of statin use								
Never	6 mo		669 (80.9)	3284 (79.4)	1.00		1.00	
Ever	6 mo							
		<0.5	52 (6.3)	301 (7.3)	0.85 (0.63‐1.15)	.29	0.69 (0.49‐0.98)	.038
		0.5‐1	27 (3.3)	154 (3.7)	0.86 (0.57‐1.31)	.48	0.60 (0.37‐0.97)	.037
		≥1	79 (9.6)	396 (9.6)	0.98 (0.76‐1.26)	.87	0.73 (0.55‐0.98)	.036

Abbreviations: CI confidence interval; DDD, defined daily dose; LT, lag time; OR, odds ratio; PDAC, pancreatic ductal adenocarcinoma.

a Adjusted age, sex, income, index year and underlying disease including DM, PCL, AP, CP, cholelithiasis.

### 3.4 Association between aspirin use and PDAC

There were 31.6% (261 of 827) “aspirin ever” users in the PDAC group, and 32.0% (1322 of 4135) in the control group without a significant difference (*P* = .838). The median aspirin exposure was 1.90 DDD‐years (interquartile range [IQR], 0.49‐4.82) in 1583 patients. (Table 4) We also calculated the aORs with 95% CIs of the association between exposure to aspirin and PDAC with adjustment of confounding factors, including DM, AP, CP, PCL, and cholelithiasis. There were no significant differences in aspirin exposure between the PDAC group and the control group. The OR was 0.98 (95% CI 0.84‐1.15; *P* = .84), and the aOR was 0.84 (95% CI 0.70‐1.01; *P* = .068). We observed no significant differences between the two groups even when other lag times were applied. According to the dose‐effect relationship analysis of aspirin that was estimated by DDD‐year, the OR was 0.98 (95% CI 0.80‐1.21; *P* = .846) and the aOR was 0.84 (95% CI 0.67‐1.07; *P* = .154) for “less than the median” user, as the OR of a “more than median” user was 1.00 (95% CI 0.81‐1.23; *P* = .971), and the aOR was 0.84 (95% CI 0.67‐1.07, *P* = .151). The ORs and aORs of three groups divided by one DDD‐year interval were as follows: 1.01 (95% CI 0.82‐1.24; *P* = .959) and 0.85 (95% CI 0.68‐1.07; *P* = .165) for less than one DDD‐years user, 1.10 (95% CI 0.78‐1.56; *P* = .581) and 0.88 (95% CI 0.60‐1.30, *P* = .519) for one to two DDD‐years user, 1.01 (95% CI 0.82‐1.25; *P* = .928) and 0.84 (95% CI 0.66‐1.06; *P* = .141) for more than two DDD‐years user.

**Table 4:** Relationship of aspirin use to PDAC

	LT	Dose (DDD‐y)	PDAC	Control	Crude		Adjusted^a^	
			(n = 827) N (%)	(n = 4135) N (%)	OR (95% CI)	*P*	OR (95% CI)	*P*
Aspirin use								
Never	6 mo		566 (68.4)	2813 (68.0)				
Ever	6 mo		261 (31.6)	1322 (32.0)	0.98 (0.84‐1.15)	.838	0.84 (0.70‐1.01)	.068
Never	1 mo		566 (68.4)	2813 (68.0)				
Ever	1 mo		261 (31.6)	1322 (32.0)	0.98 (0.84‐1.15)	.838	0.84 (0.70‐1.01)	.068
Never	1 y		575 (69.5)	2858 (69.1)				
Ever	1 y		252 (30.5)	1277 (30.9)	0.98 (0.83‐1.15)	.837	0.85 (0.71‐1.03)	.090
Cumulative dose of aspirin use								
Never	6 mo		566 (68.4)	2813 (68.0)	1.00		1.00	
Ever	6 mo							
		Less than median (<1.90)	132 (16.0)	682 (16.5)	0.98 (0.80‐1.21)	.846	0.84 (0.67‐1.07)	.154
		More than median (≥1.90)	129 (15.6)	640 (15.5)	1.00 (0.81‐1.23)	.971	0.84 (0.67‐1.07)	.151
Cumulative dose of aspirin use								
Never	6 mo		566 (68.4)	2813 (68.0)	1.00		1.00	
Ever	6 mo							
		<1	142 (17.2)	715 (17.3)	1.01 (0.82‐1.24)	.959	0.85 (0.68‐1.07)	.165
		1‐2	43 (5.2)	194 (4.7)	1.10 (0.78‐1.56)	.581	0.88 (0.60‐1.30)	.519
		≥2	129 (15.6)	640 (15.5)	1.01 (0.82‐1.25)	.928	0.84 (0.66‐1.06)	.141

Abbreviations: CI confidence interval; DDD, defined daily dose; LT, lag time; OR, odds ratio; PDAC, pancreatic ductal adenocarcinoma.

a Adjusted for age, sex, income, index year and underlying disease including DM, PCL, AP, CP, cholelithiasis.

### 3.5 Association between drug use and PDAC in patients with risk factors

We performed a subgroup analysis of patients with one or more of DM, AP, CP, PCL, and cholelithiasis for the association of aspirin use and statin use with PDAC. A total of 427 patients of the PDAC group and 829 patients of the control group had risk factors. There was no significant difference between the two groups in age over 70 years (50.8% for the PDAC group with risk factors, 56.0% for control group with risk factors; *P* = .083), male (57.6%, 60.8%; *P* = .302), high income level (50.8%, 47.8%; 0.587), index date between 2010‐2013 (60.7%, 68.2%). In terms of comorbidities, there were significant differences between two groups in DM (60.2% for the PDAC group with risk factors, 84.4% for control group with risk factors; *P* < .001), AP (20.4%, 3.6%; *P* < .001), CP (17.6%, 0.8%; *P* < .001), PCL (17.3%, 0.8%; *P* < .001), but there were no significant differences in cholelithiasis (16.6%, 14.8%; *P* = .411), alcohol‐related disease (9.8%, 9.0%; *P* = .682), COPD (3.3%, 5.1%; *P* = .193), CHB (4.4%, 4.0%; *P* = .765), and CHC (2.6%, 1.4%; *P* = .183).

Table 5 shows the results for analyses of the association between drug use and PDAC among these patients. There were 37.0% (158 of 427) “aspirin ever” users in the PDAC group with risk factors, and 54.8% (454 of 829) in the control group with risk factors with a significant statistical difference (*P* < .001). The median aspirin exposure was 2.21 DDD‐years (IQR, 0.60‐4.57) in 612 patients. The OR for aspirin use for both groups was 0.48 (95% CI 0. 37‐0.61; *P* < .001), and the aOR was 0.67 (95% CI 0.50‐0.89; *P* = .006). According to the cumulative exposure of aspirin use that was estimated by DDD‐year, the OR was 0.49 (95% CI 0.36‐0.66; *P* < .001) and the aOR was 0.65 (95% CI 0.46‐0.93; *P* = .017) for “less than the median” user, as the OR of a “more than median” user was 0.46 (95% CI 0.34‐0.63; *P* < .001), and the aOR was 0.68 (95% CI 0.48‐0.98, *P* = .036).

**Table 5:** Relationship of aspirin use or statin use to PDAC among patients with risk factors

Drug	Dose (DDD‐y)	PDAC with risk factors (N = 427)	Control with risk factors (N = 829)	Crude		Adjusted^a^	
		n (%)	n (%)	OR (95% CI)	*P*	OR (95% CI)	*P*
Statin use							
Never		319 (74.7)	507 (61.2)				
Ever		108 (25.3)	322 (38.8)	0.50 (0.38‐0.66)	<.001	0.62 (0.45‐0.84)	.002
Cumulative dose of statin use							
Never		319 (74.7)	507 (61.2)				
Ever	Less than median (<1.16 DDD‐y)	53 (12.4)	158 (19.1)	0.53 (0.37‐0.74)	<.001	0.56 (0.37‐0.84)	.005
	More than median (≥1.16 DDD‐y)	55 (12.9)	164 (19.8)	0.53 (0.37‐0.74)	<.001	0.75 (0.51‐1.10)	.135
Aspirin use							
Never		269 (63.0)	375 (45.2)				
Ever		158 (37.0)	454 (54.8)	0.48 (0.37‐0.61)	<.001	0.67 (0.50‐0.89)	.006
Cumulative dose of aspirin use							
Never		269 (63.0)	375 (45.2)				
Ever	Less than median (<2.21 DDD‐y)	80 (18.7)	224 (27.0)	0.49 (0.36‐0.66)	<.001	0.65 (0.46‐0.93)	.017
	More than median (≥2.21 DDD‐y)	78 (18.3)	230 (27.7)	0.46 (0.34‐0.63)	<.001	0.68 (0.48‐0.98)	.036

Abbreviations: CI confidence interval; DDD, defined daily dose; OR, odds ratio; PDAC, pancreatic ductal adenocarcinoma.

a Adjusted age, sex, income, index year and underlying disease including DM, PCL, AP, CP, cholelithiasis.

There were 25.3% (108 of 427) “statin ever” users in the PDAC group with risk factors, and 38.8% (322 of 829) in the control group with risk factors with a significant statistical difference (*P* < .001). The median aspirin exposure was 1.16 DDD‐years (IQR, 0.45‐2.43) in 430 patients. The OR for statin use for both groups was 0.50 (95% CI 0.38‐0.66; *P* < .001), and the aOR was 0.62 (95% CI 0.45‐0.84; *P* = .002). According to the cumulative exposure of aspirin use that was estimated by DDD‐year, the OR was 0.53 (95% CI 0.37‐0.74; *P* < .001) and the aOR was 0.56 (95% CI 0.37‐0.84; *P* = .005) for “less than the median” user, as the OR of a “more than median” user was 0.53 (95% CI 0.37‐0.74; *P* < .001), and the aOR was 0.75 (95% CI 0.51‐1.10, *P* = .135).

### 3.6 Association between exclusive or combined drug use and PDAC

We analyzed the association between exclusive or combined statin use and aspirin use among all patients and high‐risk patients to identify the additive effect. (Table 6) Among all study patients, exclusive statin use and aspirin use was shown in 6.8% (56 of 827) and 19.2% (159 of 827) in PDAC group, and 7.5% (310 of 4135) and 18.9% (781 of 4135) in control group. Combined statin and aspirin use was 12.3% (102 of 827) in PDAC group and 13.1% (541 of 4135) in control group. The exclusive use of statins (OR = 0.89; 95% CI 0.66‐1.20; *P* = .432; aOR = 0.64; 95% CI 0.45‐0.91; *P* = .012) was associated with more risk reduction than the exclusive use of aspirin. (OR = 0.99; 95% CI 0.82‐1.22; *P* = .993; aOR = 0.86; 95% CI 0.69‐1.07; *P* = .180). The combined use of statin and aspirin did not show further reduction of the risk of PDAC compared with the exclusive use of statin. (OR = 0.93; 95% CI 0.73‐1.17; *P* = .512; aOR = 0.68; 95% CI 0.52‐0.89; *P* = .005).

**Table 6:** Relationship of exclusive and combined drug use to PDAC

Drug use	Among all study patients					
	PDAC (N = 827)	Control (N = 4135)	Crude		Adjusted^a^	
	n (%)	n (%)	OR (95% CI)	*P*	OR (95% CI)	*P*
No drug use	510 (61.7)	2503 (60.5)	1.00		1.00	
Statin use	56 (6.8)	310 (7.5)	0.89 (0.66‐1.20)	.432	0.64 (0.45‐0.91)	.012
Aspirin use	159 (19.2)	781 (18.9)	0.99 (0.82‐1.22)	.993	0.86 (0.69‐1.07)	.180
Statin and aspirin use	102 (12.3)	541 (13.1)	0.93 (0.73‐1.17)	.512	0.68 (0.52‐0.89)	.005

Drug use	Among patients with risk factors					
	PDAC (N = 427)	Control (N = 829)	Crude	Adjusted^a^	Crude	Adjusted^a^
	n (%)	n (%)	OR (95% CI)	*P*	OR (95% CI)	*P*
No drug use	233 (54.6)	280 (33.8)	1.00		1.00	
Statin use	36 (8.4)	227 (27.4)	0.46 (0.30‐0.69)	<.001	0.50 (0.30‐0.82)	.006
Aspirin use	86 (20.1)	95 (11.5)	0.46 (0.34‐0.62)	<.001	0.61 (0.43‐0.87)	.006
Statin and aspirin use	72 (16.9)	227 (27.4)	0.38 (0.28‐0.52)	<.001	0.55 (0.38‐0.80)	.002

Abbreviations: CI confidence interval; OR, odds ratio; PDAC, pancreatic ductal adenocarcinoma.

a Adjusted for age, sex, income, index year and underlying disease including DM, PCL, AP, CP, cholelithiasis.

Among high risk patients, exclusive statin use and aspirin use was shown in 8.4% (36 of 427) and 20.1% (86 of 427) in PDAC group, and 27.4% (227 of 829) and 11.5% (95 of 829) in control group. Combined statin and aspirin use was 16.9% (72 of 427) in PDAC group and 27.4% (227 of 829) in control group. The exclusive use of statins (OR = 0.46; 95% CI 0.30‐0.69; *P* < .001; aOR = 0.50; 95% CI 0.30‐0.82; *P* = .006) showed a similar association of risk reduction with the exclusive use of aspirin. (OR = 0.46; 95% CI 0.34‐0.62; *P* < .001; aOR = 0.61; 95% CI 0.43‐0.87; *P* = .006). The combined use of statin and aspirin did not show further reduction of the risk of PDAC compared with the exclusive use of drugs after adjusting risk factors. (OR = 0.38; 95% CI 0.28‐0.52; *P* < .001; aOR = 0.55; 95% CI 0.38‐0.80; *P* = .002).

## 4 DISCUSSION

In this retrospective nested case‐control study in a nationwide cohort, we evaluated 827 PDAC cases and 4135 control cases with matching variables including age, sex, income and the index date at a 1:5 ratio. We found that statin use was significantly associated with a reduced risk of PDAC incidence of about 30% after correcting confounding factors compared to that of the nonusers, while aspirin use was not associated with the prevention of PDAC even after correcting confounding factors. Association of the amount of statin use with the prevention of PDAC was also identified after correction of confounding factors. DM, CP, AP, PCLs, and cholelithiasis were revealed as risk factors for PDAC. In subgroup analysis of patients with these risk factors, not only use of statin, but also use of aspirin showed associated with a reduced risk of PDAC incidence. The combined use of statin and aspirin did not show further reduction of the risk of PDAC compared with the exclusive use of drugs.

The risk factors for PDAC identified in this study may or may not be in line with previous studies. The risk of DM for PDAC in this study was similar to that in previous studies that reported a twofold risk, which was explained on the basis of a mechanism with insulin resistance.^27^ Chronic pancreatitis and PCLs has an incredibly high risk for PDAC in this study, which was in line with the results of recent studies that reported an almost 20‐fold risk.^28^, ^29^ Inflammatory activation of pancreatic stellate cells may play an important role in PDAC arise from underlying CP, but this progression has not been fully elucidated.^28^ Acute pancreatitis has also recently been reported to have almost a threefold risk in large‐scale population‐based studies, but the definite causality of AP is less clear,^4^, ^30^ and a much higher risk was observed in this study. Some studies also reported that cholelithiasis has about a 1.5‐fold risk of PDAC, and the researchers suggested that inflammation caused by gallstone pancreatitis may explain this association.^6^, ^31^ Risk factors in this study have higher numerical values than in previous studies mainly because of the relatively lower prevalence rate of those factors except for DM and choledocholithiasis. Alcohol‐related disease or COPD, which was analyzed as a surrogate marker of alcohol or smoking, did not increase the risk, whereas a risk of 1.5 times was reported in previous studies on smoking.^32^

Statin use was significantly associated with a reduced risk of PDAC incidence in this study, as is consistent with several studies.^33^, ^34^, ^35^ One case‐control study has shown that statin use of more than 6 months was associated with a risk reduction of pancreatic cancer and a dose‐response relationship was confirmed.^33^ Another case‐control study reported that statin use was associated with about 40% reduced PDAC risk, but concomitant statin and aspirin use did not further reduce the risk compared with statin use alone and no interaction was evident.^34^ A meta‐analysis for statin use revealed a significant decrease in pancreatic cancer risk by 0.84 times,^12^ which was similar to the results of this study, but the results should be interpreted conservatively, because of the significant heterogeneity between the included studies. On the other hand, several studies showed no association between statin use and the risk of PDAC.^36^, ^37^, ^38^ A population‐based case‐control study with claim data showed no beneficial association between usage of statin and PDAC after adjustment of confounding factors including DM, CP, hospitalization, and other lipid lowering drugs.^38^ One randomized controlled study showed no difference in the incidence of pancreatic cancer in comparison with a placebo group,^36^ but only 19 PDAC patients were included in this study despite a long‐term follow‐up period. A recent prospective cohort study published a combined analysis of two large cohorts, and reported that the use of statins was independent of the risk of PDAC, regardless of exposure period.^37^

The association between aspirin use and prevention for PDAC remains unclear because previous studies reported inconsistent conclusions. Several studies showed no definite association between aspirin use and prevention for PDAC, which have been consistent with our findings,^20^, ^39^, ^40^, ^41^, ^42^ but other studies have provided some support that aspirin use might reduce risk of developing pancreatic cancer.^21^, ^43^, ^44^, ^45^ Recently, a prospective cohort study has reported that regular use of aspirin does not inhibit PDAC.^20^ A multicenter hospital‐based case–control study for long‐term use with more than 5‐years of aspirin reported might have a chemoprevention effect,^45^ but the part of the results of two large cohort studies have shown no difference for even such long‐term users.^41^, ^42^ In a recent published meta‐analysis consisted with 7 case‐control studies and 7 cohort studies, the aspirin use showed a protective effect on PDAC (relative risk = 0.80, 95% CI 0.68‐0.93),^46^ although significant heterogeniety was observed and the results of subgroup analyses of studies were included in this meta‐analysis.

We conducted a subgroup analysis of patients with risk factors identified in this study, and found that both aspirin use and statin use were associated about 40% reduced PDAC risk after correcting the confounding factors. Although this subgroup was influenced by selection bias because the patients in both groups were not selected in the prematching level, there was no significant difference in age, sex, income and index year in both groups. Recently, well‐designed studies with a large number of patients have reported the relationship between statin use and PDAC in high risk patients with DM or CP. Statin use significantly decreased the risk of pancreatic cancer in nearly 50% in patients with type 2 DM,^47^ but that for patients with CP was not clear because two previous studies reported inconsistent conclusions.^48^, ^49^ Meanwhile, a nearly 30% lower risk for pancreatic cancer with regular aspirin use among participants with DM was observed in a large cohort study,^20^ which was similar to result of this study, but it does not appear to be affected by the cumulative dose of aspirin use. Few studies have been conducted on the relationship between aspirin use and PDAC for patients with risk factors, and it is necessary to further evaluate in the future.

This study has several strengths. First, we conducted this case‐control study in NHIS‐NSC, which was proven to be representative of the nationwide 12‐year Korean population and could minimize selection bias, because the Republic of Korea has a compulsory universal health insurance system by NHIS. Also, we tried to minimize the selection bias in the baseline characteristics by matching age, sex, income, and index year. Second, we did a subgroup analysis on the association between drug use and the risk of PDAC among the patients with risk factors for PDAC including DM, PCLs, AP, CP, and cholelithiasis, which have not been sufficiently considered in previous studies. Finally, various efforts have been made to accurately assess information on drug exposure. Accurate and quantitative analyses of prescription records for drugs were possible using the overall claims data from all outpatient clinics and medical institutions in the NHIS‐NSC data. In addition, drug exposures were reliably assessed by using the validated DDD system with at least 5 years of review for each patient to accurately measure the effect of exposure, and all patients had at least one drug‐free year. The inclusion of only those patients who received at least 30 DDDs of prescriptions as an exposure group to confirm the effectiveness of certain exposures as compared to other studies also reduced bias.

This study showed several limitations which should be addressed in further studies. First, a certain level of inaccuracy comes from inferring the causal relationship through an operational definition of the disease, because of the discrepancies between claims data and real practice. We attempted to define the disease in this study to prevent overestimation of diseases by using admission to hospital, an imaging test, or a disease‐specific drug in addition to the ICD‐10 code. Second, selection bias of this case‐control study comes from its nature as an observation study. However, we believe that the selection bias might be lower than a traditional case‐control study because the PDAC and control patients were selected from a population‐based nation‐wide cohort. Third, there is a limitation in consideration of risk factors because the NHIS‐NSC has details on exposure to smoking, alcohol, and health checkup data for only a few selected patients. To compensate for the lack of tobacco exposure data, we considered COPD as a surrogate marker for patients' exposure to smoking in this study. Fourth, it might be difficult to interpret the results in the subgroup analysis according to risk factors because selection bias could have existed, although there was no difference in baseline characteristics between the two groups. In addition, drug exposure in the real world may not be included in the claims data, because we do not know how much compliance the patient had with the drug in the real world, which may result in overestimation of medication use under certain conditions. We carried out sensitivity analyses by changing the lag period and the drug exposure, and the results were consistent. Finally, there was a limit to conclusions on dose‐effectiveness or long‐term outcomes, because the median drug exposure were revealed about 2 years for aspirin and about 1 year for statin.

In conclusion, the results of this nested case‐control study suggests that statin use was associated with a reduced risk of PDAC incidence but aspirin use was not. In patients with risk factors, aspirin use and statin use were associated a reduced risk of PDAC incidence, but it is not reasonable to draw conclusions due to selection bias. It is likely that a further well‐designed large‐scale prospective cohort or randomized controlled trial would provide definitive evidence. Also, the mechanism of aspirin use or statin use and PDAC development should be addressed in further animal and human clinical trials.

## CONFLICT OF INTEREST

None declared.

## AUTHOR CONTRIBUTIONS

Jin Ho Choi planned and conducted the study, collected and interpreted the data, was involved in the statistical analysis, and drafted the manuscript. He approved the final draft for submission. Sang Hyub Lee planned and conducted the study, critically revised the manuscript for important intellectual content, and supervised the study. He approved the final draft for submission. Gunn Huh collected and interpreted data, was involved in the statistical analysis, and drafted the manuscript. He approved the final draft for submission. Jung Won Chun collected and interpreted the data and drafted the manuscript. He approved the final draft for submission. Min Su You collected and interpreted data and drafted the manuscript. He approved the final draft for submission. Woo Hyun Paik planned the study and critically revised the manuscript for important intellectual content. He approved the final draft for submission. Ji Kon Ryu critically revised the manuscript for important intellectual content, and supervised the study. He approved the final draft for submission. Yong‐Tae Kim critically revised the manuscript for important intellectual content and supervised the study. He approved the final draft for submission.

## Data Availability

The datasets used and/or analyzed in the study are available from the corresponding author on reasonable request.
